# Supplementary material for: The Anti-Tubercular Aminolipopeptide Trichoderin A Displays Selective Toxicity against Human Pancreatic Ductal Adenocarcinoma Cells Cultured under Glucose Starvation
Source: Pharmaceutics. 2023 Jan 14;15(1):287. doi: 10.3390/pharmaceutics15010287 (PMC9866366; doi:10.3390/pharmaceutics15010287)
Supplement: Supplementary file 1 [file pharmaceutics-15-00287-s001.zip › pharmaceutics-2128652-supplementary.pdf]

Supporting information for:

The Anti-Tubercular Aminolipopeptide Trichoderin A Displays Selective Toxicity against Human Pancreatic Ductal Adenocarcinoma Cells Cultured under Glucose Starvation

Johanes K. Kasim [1,2], Jiwon Hong [1,2,3,4], Anthony J. R. Hickey [1,2], Anthony R. J. Phillips [1,2,3,4], John A. Windsor [2,3,4],
Paul W. R. Harris [1,2,5], Margaret A. Brimble [1,2,5] and Iman Kavianinia [1,2,5,*]

[1] School of Biological Sciences, The University of Auckland, 3A Symonds Street, Auckland 1010, New Zealand
[2] Maurice Wilkins Centre for Molecular Biodiscovery, The University of Auckland, 3A Symonds Street, Auckland 1010, New Zealand
[3] Surgical and Translational Research Centre, The University of Auckland, 22-30 Park Avenue, Auckland 1023, New Zealand
[4] Department of Surgery, The University of Auckland, 22-30 Park Avenue, Auckland 1023, New Zealand
[5] School of Chemical Sciences, The University of Auckland, 23 Symonds Street, Auckland 1010, New Zealand
* Correspondence: i.kavianinia@auckland.ac.nz

Materials and Methods

Materials

All reagents purchased from commercial vendors were used without additional purification. *N,N*-diisopropylethylamine (DIPEA; ReagentPlus grade), piperidine, propionic acid, butanoic acid, hexanoic acid, octanoic acid, decanoic acid, dodecanoic acid, formic acid (LC-MS grade), acetic anhydride, *N,N′*-diisopropylcarbodiimide (DIC), and dimethyl sulfoxide (DMSO, cell culture grade) were supplied by Sigma-Aldrich (St Louis, MO). *N,N*-dimethylformamide (DMF), dichloromethane (CH_2Cl_2), and methanol (MeOH) were purchased from ECP Ltd. (Auckland, NZ). Milli-Q water (H_2O) for RP-HPLC and LC-MS was obtained from the Sartorius arium pro ultrapure water production system (Gottingen, Germany). *O*-(7-azabenzotriazol-1-yl)-*N,N,N′,N′*-tetramethyluronium hexafluorophosphate (HATU) and ethyl-2-cyano-2-(hydroxyamino)acetate (Oxyma Pure) were purchased from Novabiochem (Darmstadt, Germany). 1-[(1-(Cyano-2-ethoxy-2-oxoethylideneaminooxy)-dimethylaminomorpholinomethylene)] methanaminium hexafluorophosphate (COMU) and 6-chloro-1-hydroxybenzotriazole (6-Cl-HOBt) were sourced from Aapptec (Louisville, KY). HPLC and LC-MS grade acetonitrile (CH_3CN) were purchased from Merck (Darmstadt, Germany). Trifluoroacetic acid (TFA) was purchased from Oakwood Chemicals (Estill, SC). 2-Chlorotrityl chloride (2-CTC)

polystyrene resin, Fmoc-α-aminoisobutyric acid (Aib), Fmoc-Ile-OH, Fmoc-Val-OH, and Fmoc-Pro-OH were purchased from CS Bio (Shanghai, China). Boc-Pro-OH was purchased from PolyPeptide Group (Zug, Switzerland). Fmoc-Cha-OH, and 1,1,1,3,3,3-hexafluoro-2-propanol (HFIP) were purchased from AK Scientific (Union City, CA). Leucinostatin A was purchased from Santa Cruz Biotechnology, Inc. (Dallas, TX). Trichoderin A, (2*R*)-methyldecanoic acid (MDA), (2*S*,4*S*,6*R*)-2-amino-6-hydroxy-4-methyl-8-oxodecanoic acid (AHMOD) and (*S*)-2-(2-aminopropyl(methyl)amino)ethanol (AMAE) were synthesised in house as previously reported [1]. Nunc MicroWell 96-Well Black Optical-Bottom Plates with Polymer Base, PrestoBlue™ Cell Viability Reagent, CyQUANT™ Direct Cell Proliferation Assay, Roswell Park Memorial Institute (RPMI) 1640 medium (ATCC Modification, #A1049101; 25 mM glucose), RPMI 1640 medium (no glucose, #11879020), Dulbecco's Eagle Modified Medium (DMEM; high glucose, pyruvate, #11995040; 25 mM glucose), DMEM (no glucose, #11966025), sodium pyruvate (100 mM), d-glucose solution (200 g/L), antibiotic-antimycotic (A/A), TrypLE™ Express Enzyme (1×) (phenol red, #12605010), and phosphate-buffered saline (PBS, pH 7.4) were purchased from Thermo Fisher Scientific (Waltham, MA). Foetal bovine serum (FBS, heat-inactivated) was purchased from Moregate Biotech (Hamilton, NZ).

General Procedure for Peptide Synthesis

The peptides were assembled manually by 9-fluorenylmethoxycarbonyl/*tert*-butyl solid phase peptide synthesis (Fmoc/*t*Bu SPPS) in a fritted glass reaction vessel.

<u>Method 1) Attachment of Fmoc-Aib-OH on resin</u>

A solution of Fmoc-Aib-OH (5 eq., 162.7 mg, 0.5 mmol) and DIPEA (10 eq., 172 µL, 1 mmol) in CH_2Cl_2 (3 mL) was added to 2-CTC resin (112.4 mg, 0.1 mmol; loading: 0.89 mmol/g) that has been pre-swollen in CH_2Cl_2 (3 mL) for 15 min. The reaction mixture was agitated for 6 h at room temperature, filtered, and repeated with fresh reagents for a further 6 h. Excess solvent was then filtered, and the resin bed treated with a mixture of CH_2Cl_2/MeOH/DIPEA (8:1.5:0.5, *v*/*v*/*v*, 3 mL) for 15 min at room temperature to cap unreacted chlorides on the resin.

<u>Method 2) Spectrophotometric quantitation of first residue loading on resin</u>

Loading of the first residue on resin was quantified on the Shimadzu UV-1280 UV-VIS spectrophotometer (Kyoto, Japan). Resin that has been dried under vacuum was weighed into a Starna Scientific 10-mm matched silica UV spectrophotometric cuvette (Ilford, UK). 20% piperidine/DMF (*v*/*v*, 3 mL) was dispensed into the cuvette, briefly agitated with a pipettor to achieve a uniform suspension, then left for 5 min at room temperature. A reference cuvette, containing only 20% piperidine/DMF (*v*/*v*, 3 mL), was used to zero the spectrophotometer at the wavelength of 290 nm. The absorbance

of the resin-containing cuvette was recorded as an average of three independent measurements, and the loading of the first residue calculated by the following **Equation (1)**:

$$Loading = \frac{Absorbance}{(Resin\ mass\ \times 1.75)}$$

Equation (1). Estimation of first residue attachment on resin. Loading is measured in mmol/g.

Method 3) Removal of Fmoc protecting group

A solution of 20% piperidine/DMF (*v/v*, 3 mL) was added to the peptidyl resin to remove the Fmoc protecting group. The reaction mixture was agitated for 5 min at room temperature, filtered, and the reaction repeated with fresh reagents for a further 10 min. Excess solvent was then filtered and the resin bed washed with DMF (3 × 3 mL).

Method 4) Procedure for the difficult sequential Aib-Aib coupling on resin

A solution of Fmoc-Aib-OH (5 eq., 162.7 mg, 0.5 mmol), COMU (5 eq., 214 mg, 0.5 mmol), Oxyma (5 eq., 71 mg, 0.5 mmol) and DIPEA (10 eq., 172 µL, 1 mmol) in DMF (3 mL) was added to the peptidyl resin (0.1 mmol). The reaction mixture was agitated for 2 h at room temperature, filtered, and the reaction repeated with fresh reagents for a further 2 h. Excess solvent was then filtered and the resin bed washed with DMF (3 × 3 mL).

Method 5) Coupling of Fmoc-protected amino acid

A solution of the appropriate Fmoc-Aa-OH (5 eq., 0.5 mmol), HATU (4.9 eq., 186 mg, 0.49 mmol) and DIPEA (10 eq., 172 µL, 1 mmol) in DMF (3 mL) was added to the peptidyl resin (0.1 mmol). The reaction mixture was agitated for 1 h at room temperature, filtered, and the reaction repeated with fresh reagents for a further 1 h. Excess solvent was then filtered and the resin bed washed with DMF (3 × 3 mL).

Method 6) Attachment of N-terminal fatty acid

A solution of the appropriate fatty acid (5 eq., 0.5 mmol), COMU (5 eq., 214 mg, 0.5 mmol), Oxyma (5 eq., 71 mg, 0.5 mmol) and DIPEA (10 eq., 172 µL, 1 mmol) in DMF (3 mL) was added to the peptidyl resin (0.1 mmol). The reaction mixture was agitated for 3 h at room temperature. Excess solvent was then filtered, the resin bed washed with DMF (3 × 3 mL) and CH_2Cl_2 (3 × 3 mL), then dried under vacuum.

Method 7) HFIP-mediated resin cleavage

The resin bed was treated with 20% HFIP/CH_2Cl_2 (*v*/*v*, 5 mL) to release the completed peptide chain. The reaction mixture was agitated for 30 min at room temperature, filtered, and the reaction repeated with fresh reagents for a further 30 min. The collected filtrate was partially concentrated under a gentle stream of nitrogen, reconstituted in CH_3CN/H_2O (*v*/*v*, 10 mL) and lyophilised.

Method 8) Late-stage solution phase C-terminal coupling of AMAE

The *C*-terminal AMAE residue was conjugated to the peptide as a TFA salt. A solution of AMAE.2TFA (3 eq.), DIC (6 eq.), 6-Cl-HOBt (6 eq.), and DIPEA (6 eq.) in DMF was added to the purified peptide from **Method 7**. The reaction mixture was agitated for 12 h at room temperature and completion of the reaction was monitored by analytical RP-HPLC and ESI-MS. The peptide mixture was then purified batch-wise by semi-preparative RP-HPLC.

Synthesis of trichoderin A analogue (12)

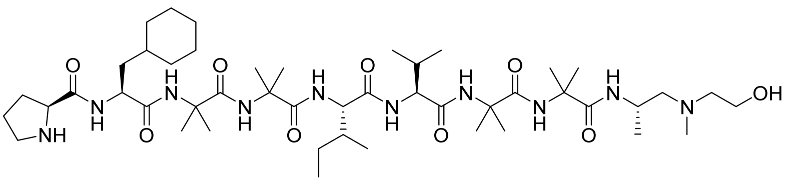

Figure S1. Chemical structure of trichoderin A analogue **(12)**.

The *C*-terminal residue Fmoc-Aib-OH was attached to 2-chlorotrityl chloride resin **S1** according to **Method 1** to form peptidyl resin **S2** (**Scheme S1**). Loading was determined to be 0.43 mmol/g by **Method 2**. The N^{α}-Fmoc-protecting group was removed using **Method 3** followed by coupling of Fmoc-Aib-OH via **Method 4** to give **S3**. The N^{α}-Fmoc-protecting group was then removed using **Method 3** followed by coupling of Fmoc-Val-OH, Fmoc-Ile-OH, and Fmoc-Aib-OH via **Method 5** to afford peptidyl resin **S4**. After removal of N^{α}-Fmoc-protecting group using **Method 3**, Fmoc-Aib-OH was coupled via **Method 4** to yield **S5**, followed by coupling of Fmoc-Cha-OH and Boc-Pro-OH by **Method 5** to complete the linear peptide sequence of **S6**. Resin-bound peptide was cleaved using **Method 7**, and the crude residue was purified by semi-preparative RP-HPLC using conditions outlined in the general method. Fractions were collected at 1 min intervals and analysed by ESI-MS and RP-HPLC. Fractions identified with the correct *m*/*z* were combined and lyophilised to afford peptide **S7** as white fluffy flakes (27.9 mg, 30.3% yield). Late-stage solution phase *C*-terminal coupling was employed to introduce the *C*-terminal aminoalcohol moiety on the peptide (27.9 mg, 0.03 mmol) dissolved in 0.5 mL DMF using **Method 8**. The crude peptide solution was purified by semi-preparative RP-HPLC in 5 × 100 μL batches using conditions outlined in the general method. Fractions were collected at 1 min intervals and analysed by ESI-MS and RP-HPLC. Fractions identified with the correct *m*/*z* were combined, lyophilised, and subjected to N^{α}-Boc deprotection using TFA/CH_2Cl_2 (1:1, *v*/*v*) at room temperature for 1 h to afford trichoderin A analogue **(12)** as a white amorphous solid (7.6 mg, 8.1% overall yield based on 0.1 mmol resin loading, 98.0% purity); t_R 16.0 min; LRMS: *m*/*z* (ESI-MS) 935.2 ($[M+H]^+$ requires 935.3), **Figures S1-S2**. The peptide was subsequently evaluated for its *in vitro* cytotoxicity against BxPC-3 and PANC-1 human PDAC cells under both glucose-replete and glucose-deprived conditions on the same plate (**Figure S3**).

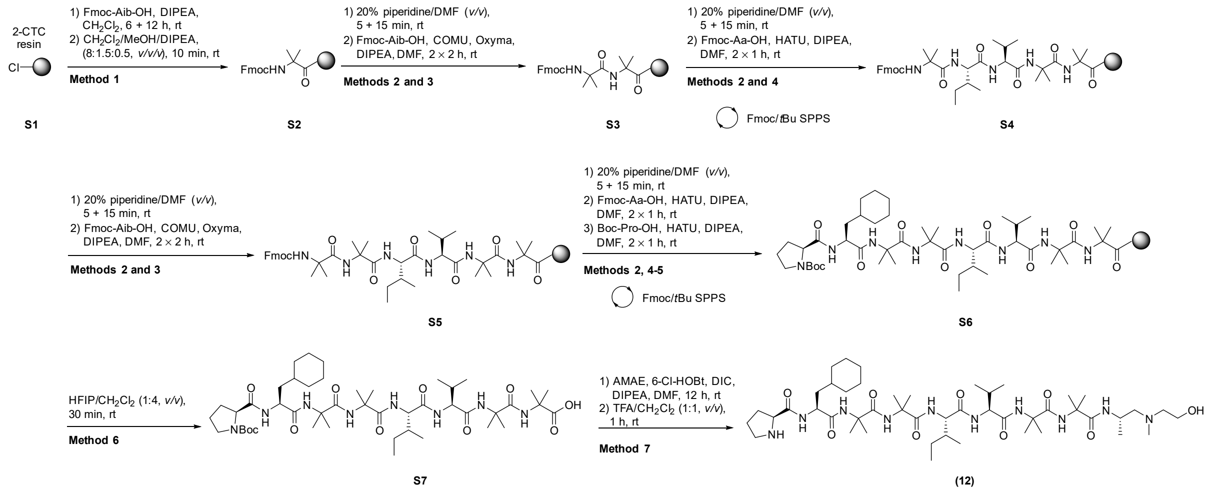

Scheme S1. Fmoc/*t*Bu SPPS of trichoderin A analogue **(12)**.

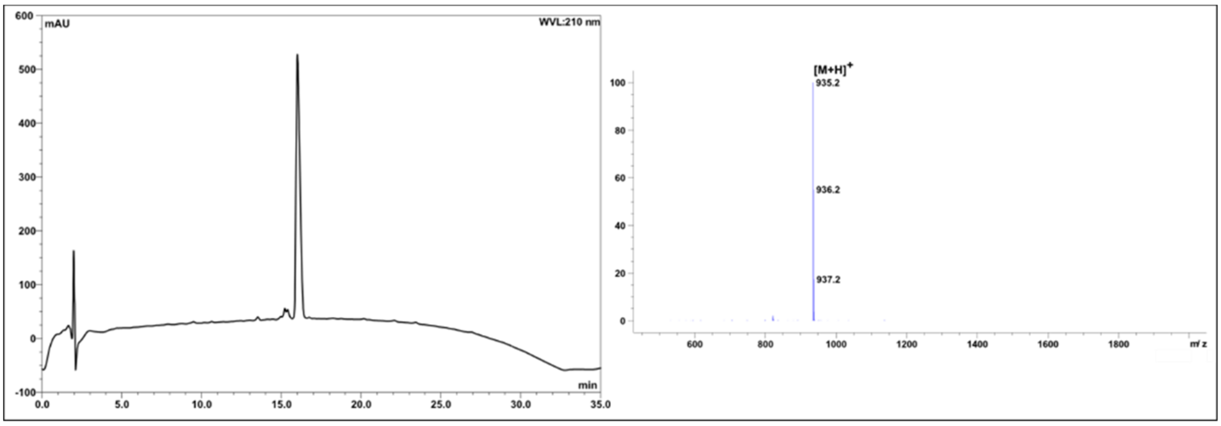

Figure S2. Analytical RP-HPLC profile of pure peptide **(12)** (*ca*. 98% as judged by peak area at 210 nm); xTerra® MS C18, 4.6 × 150 mm, 5 µm, linear gradient of 5% B to 95% B (*ca*. 3% B/min), 1 mL/min. *m*/*z* $[M+H]^+$ calculated: 935.3; found: 935.2.

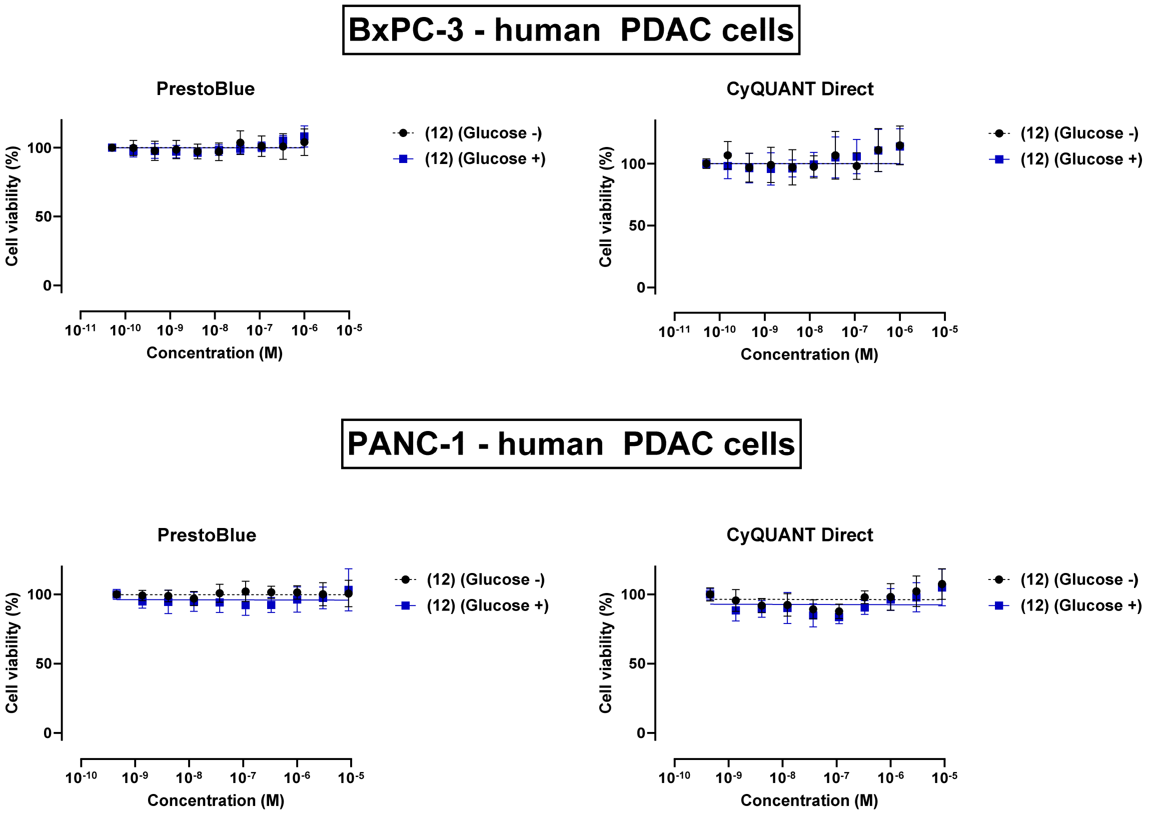

Figure S3. Comparative percent viability of BxPC-3 and PANC-1 human PDAC cells cultured under 'low glucose' (Glucose -) and 'normal glucose' (Glucose +) conditions following a 1-day exposure to trichoderin A analogue **(12)**, as judged by the PrestoBlue™ and CyQUANT™ Direct assays.

Synthesis of trichoderin A analogue (13)

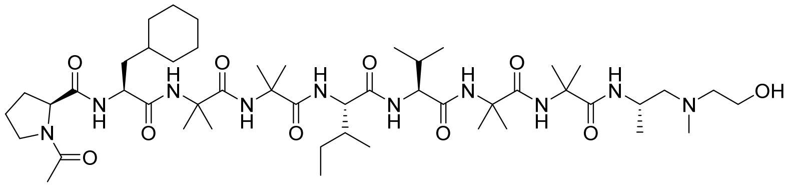

Figure S4. Chemical structure of trichoderin A analogue **(13)**.

The *C*-terminal residue Fmoc-Aib-OH was attached to 2-chlorotrityl chloride resin **S1** according to **Method 1** to form peptidyl resin **S2** (**Scheme S2**). Loading was determined to be 0.43 mmol/g by **Method 2**. The N^{α}-Fmoc-protecting group was removed using **Method 3** followed by coupling of Fmoc-Aib-OH via **Method 4** to give **S3**. The N^{α}-Fmoc-protecting group was then removed using **Method 3** followed by coupling of Fmoc-Val-OH, Fmoc-Ile-OH, and Fmoc-Aib-OH via **Method 5** to afford peptidyl resin **S4**. After removal of N^{α}-Fmoc-protecting group using **Method 3**, Fmoc-Aib-OH was coupled via **Method 4** to yield **S5**, followed by coupling of Fmoc-Cha-OH and Fmoc-Pro-OH by **Method 5**. The N^{α}-Fmoc protecting group was then removed via **Method 3** and the *N*-terminus capped with 20% acetic anhydride/DMF (*v*/*v*) at room temperature for 2 × 5 min to complete the linear peptide sequence of **S8**. Resin-bound peptide was cleaved using **Method 7**, and the crude residue was purified by semi-preparative RP-HPLC using conditions outlined in the general method. Fractions were collected at 1 min intervals and analysed by ESI-MS and RP-HPLC. Fractions identified with the correct *m*/*z* were combined and lyophilised to afford peptide **S9** as white fluffy flakes (7.2 mg, 8.3% yield). Late-stage solution phase *C*-terminal coupling was employed to introduce the *C*-terminal aminoalcohol moiety on the peptide (7.6 mg, 0.009 mmol) dissolved in 0.5 mL DMF using **Method 8**. The crude peptide solution was purified by semi-preparative RP-HPLC in 5 × 100 μL batches using conditions outlined in the general method. Fractions were collected at 1 min intervals and analysed by ESI-MS and RP-HPLC. Fractions identified with the correct *m*/*z* were combined and lyophilised to afford trichoderin A analogue **(13)** as a white amorphous solid (4.0 mg, 4.1% overall yield based on 0.1 mmol resin loading, >99% purity); t_R 20.7 min; LRMS: *m*/*z* (ESI-MS) 977.2 ($[M+H]^+$ requires 977.3), **Figures S4-S5**. The peptide was subsequently evaluated for its *in vitro* cytotoxicity against BxPC-3 and PANC-1 human PDAC cells under both glucose-replete and glucose-deprived conditions on the same plate (**Figure S6**).

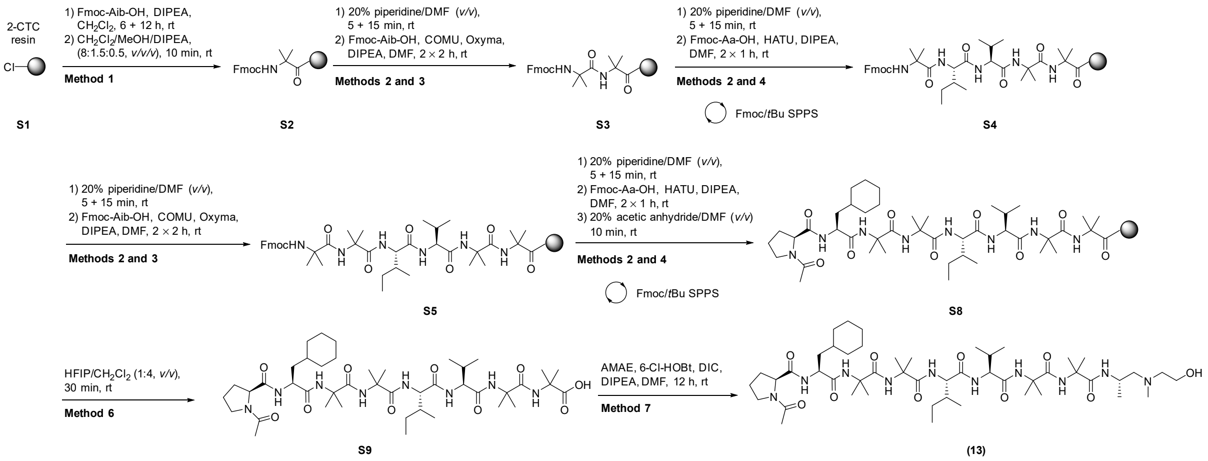

Scheme S2. Fmoc/*t*Bu SPPS of trichoderin A analogue **(13)**.

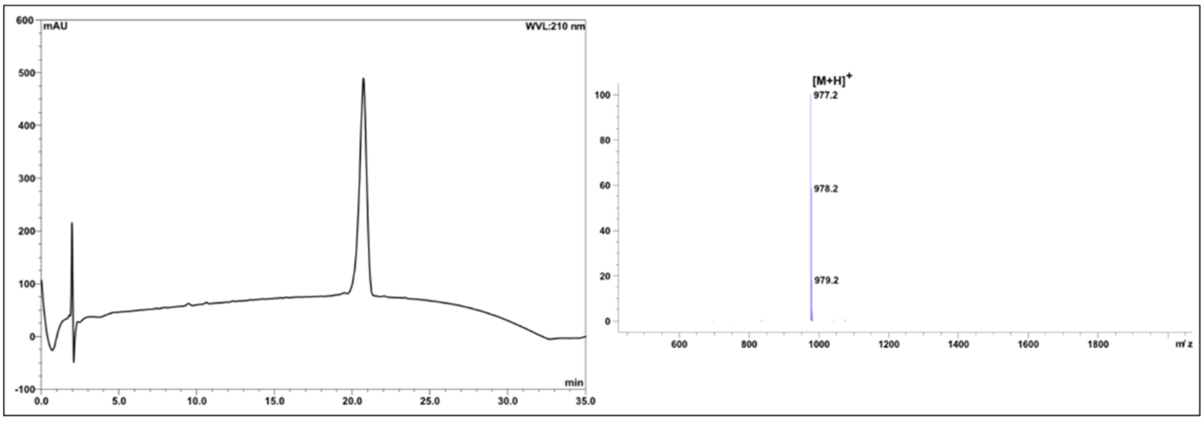

Figure S5. Analytical RP-HPLC profile of pure peptide **(13)** (*ca*. 99% as judged by peak area at 210 nm); xTerra® MS C18, 4.6 × 150 mm, 5 µm, linear gradient of 5% B to 95% B (*ca*. 3% B/min), 1 mL/min. *m*/*z* [M+H]$^+$ calculated: 977.3; found: 977.2.

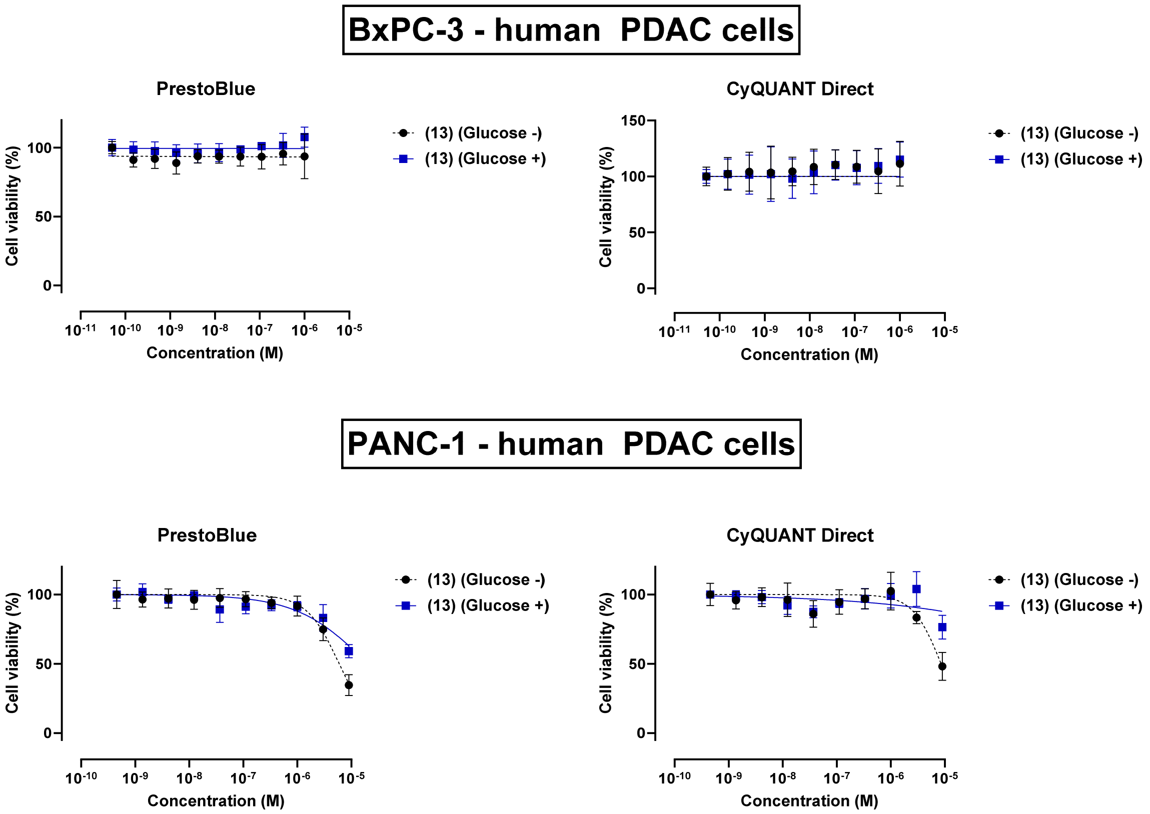

Figure S6. Comparative percent viability of BxPC-3 and PANC-1 human PDAC cells cultured under 'low glucose' (Glucose -) and 'normal glucose' (Glucose +) conditions following a 1-day exposure to trichoderin A analogue **(13)**, as judged by the PrestoBlue™ and CyQUANT™ Direct assays.

Synthesis of trichoderin A analogue (14)

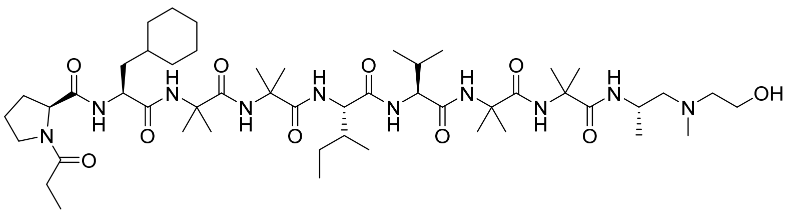

Figure S7. Chemical structure of trichoderin A analogue **(14)**.

The *C*-terminal residue Fmoc-Aib-OH was attached to 2-chlorotrityl chloride resin **S1** according to **Method 1** to form peptidyl resin **S2** (**Scheme S3**). Loading was determined to be 0.43 mmol/g by **Method 2**. The N^{α}-Fmoc-protecting group was removed using **Method 3** followed by coupling of Fmoc-Aib-OH via **Method 4** to give **S3**. The N^{α}-Fmoc-protecting group was then removed using **Method 3** followed by coupling of Fmoc-Val-OH, Fmoc-Ile-OH, and Fmoc-Aib-OH via **Method 5** to afford peptidyl resin **S4**. After removal of N^{α}-Fmoc-protecting group using **Method 3**, Fmoc-Aib-OH was coupled via **Method 4** to yield **S5**, followed by coupling of Fmoc-Cha-OH and Fmoc-Pro-OH by **Method 5**, and propionic acid via **Method 6** to complete the linear peptide sequence of **S10**. Resin-bound peptide was cleaved using **Method 7**, and the crude residue was purified by semi-preparative RP-HPLC using conditions outlined in the general method. Fractions were collected at 1 min intervals and analysed by ESI-MS and RP-HPLC. Fractions identified with the correct *m/z* were combined and lyophilised to afford peptide **S11** as white fluffy flakes (27.2 mg, 31.0% yield). Late-stage solution phase *C*-terminal coupling was employed to introduce the *C*-terminal aminoalcohol moiety on the peptide (27.2 mg, 0.03 mmol) dissolved in 0.5 mL DMF using **Method 8**. The crude peptide solution was purified by semi-preparative RP-HPLC in 5 × 100 μL batches using conditions outlined in the general method. Fractions were collected at 1 min intervals and analysed by ESI-MS and RP-HPLC. Fractions identified with the correct *m/z* were combined and lyophilised to afford trichoderin A analogue **(14)** as a white amorphous solid (10.2 mg, 10.3% overall yield based on 0.1 mmol resin loading, 98.9% purity); t_R 21.5 min; LRMS: *m/z* (ESI-MS) 991.2 ($[M+H]^+$ requires 991.3), **Figures S7-S8**. The peptide was subsequently evaluated for its *in vitro* cytotoxicity against BxPC-3 and PANC-1 human PDAC cells under both glucose-replete and glucose-deprived conditions on the same plate (**Figure S9**).

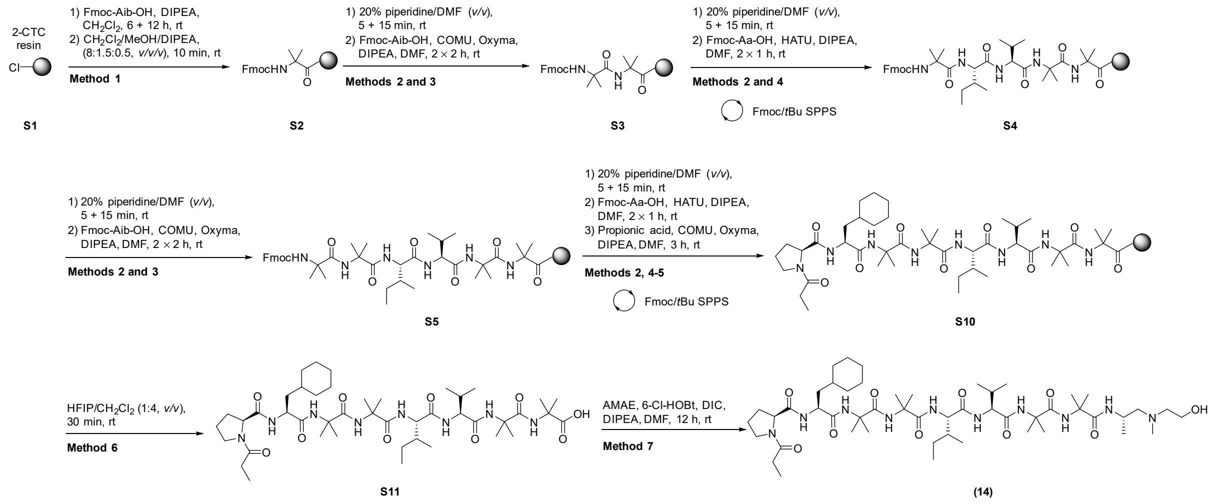

Scheme S3. Fmoc/*t*Bu SPPS of trichoderin A analogue **(14)**.

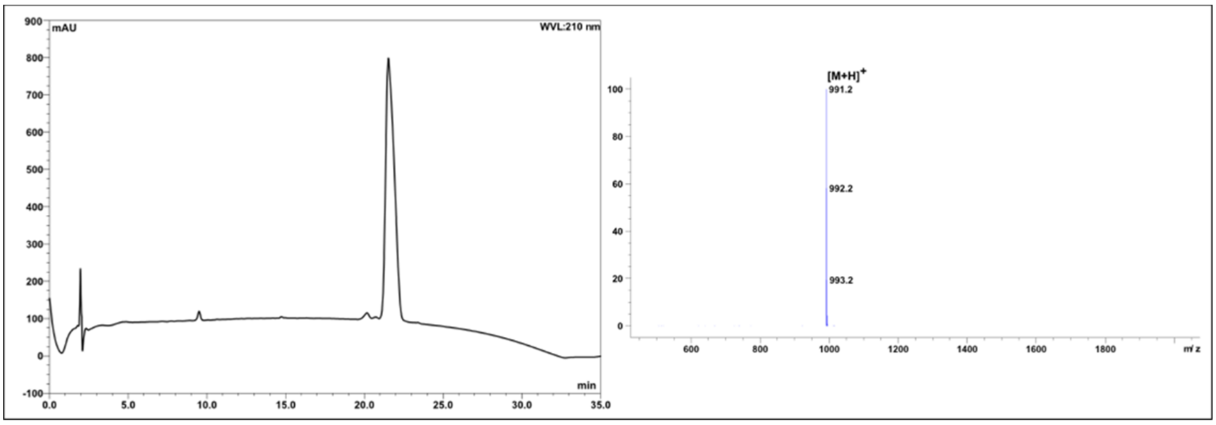

Figure S8. Analytical RP-HPLC profile of pure peptide **(14)** (*ca*. 99% as judged by peak area at 210 nm); xTerra® MS C18, 4.6 × 150 mm, 5 μm, linear gradient of 5% B to 95% B (*ca*. 3% B/min), 1 mL/min. *m*/*z* $[M+H]^+$ calculated: 991.3; found: 991.2.

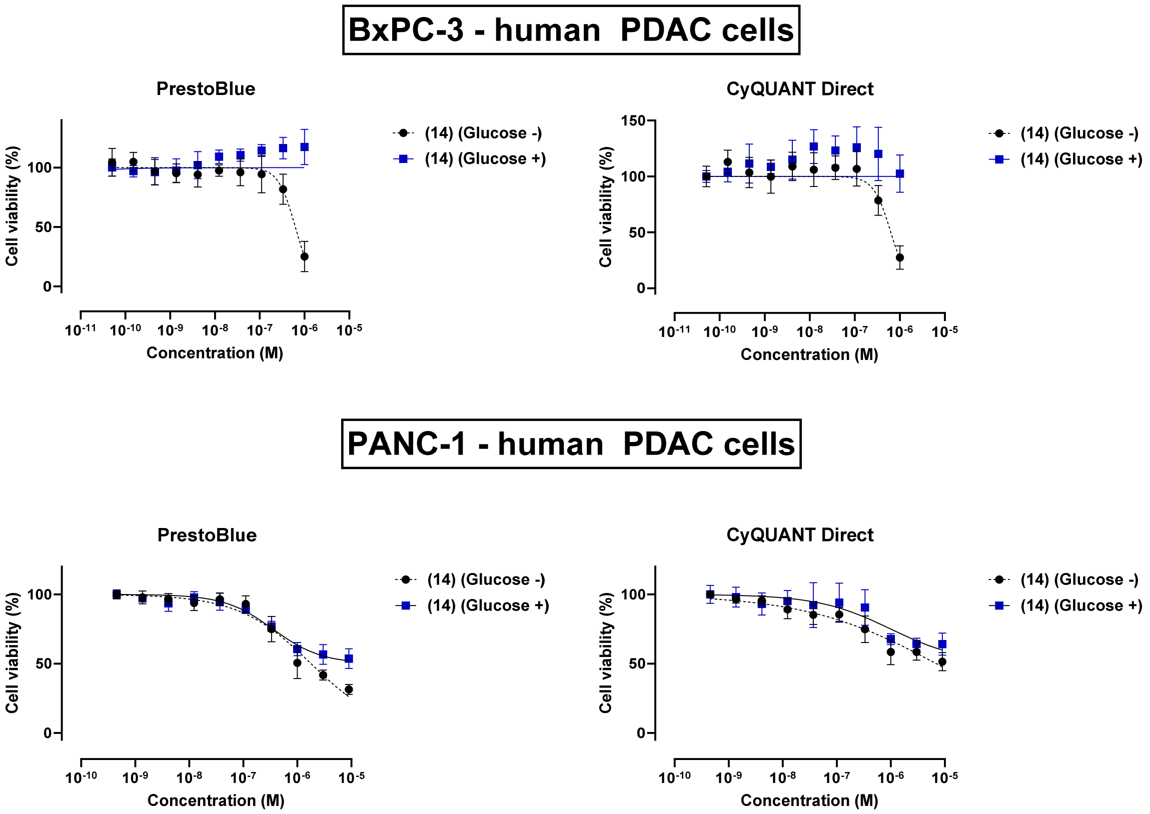

Figure S9. Comparative percent viability of BxPC-3 and PANC-1 human PDAC cells cultured under 'low glucose' (Glucose -) and 'normal glucose' (Glucose +) conditions following a 1-day exposure to trichoderin A analogue **(14)**, as judged by the PrestoBlue™ and CyQUANT™ Direct assays.

Synthesis of trichoderin A analogue (15)

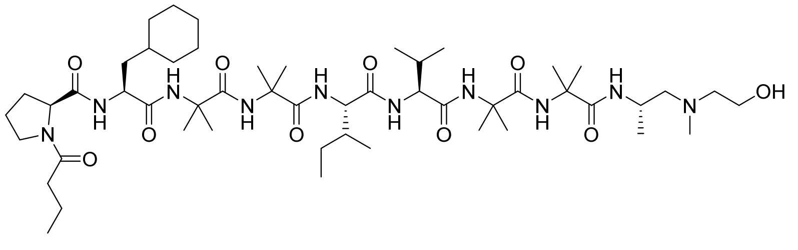

Figure S10. Chemical structure of trichoderin A analogue **(15)**.

The *C*-terminal residue Fmoc-Aib-OH was attached to 2-chlorotrityl chloride resin **S1** according to **Method 1** to form peptidyl resin **S2** (**Scheme S4**). Loading was determined to be 0.43 mmol/g by **Method 2**. The N^{α}-Fmoc-protecting group was removed using **Method 3** followed by coupling of Fmoc-Aib-OH via **Method 4** to give **S3**. The N^{α}-Fmoc-protecting group was then removed using **Method 3** followed by coupling of Fmoc-Val-OH, Fmoc-Ile-OH, and Fmoc-Aib-OH via **Method 5** to afford peptidyl resin **S4**. After removal of N^{α}-Fmoc-protecting group using **Method 3**, Fmoc-Aib-OH was coupled via **Method 4** to yield **S5**, followed by coupling of Fmoc-Cha-OH and Fmoc-Pro-OH by **Method 5**, and butyric acid via **Method 6** to complete the linear peptide sequence of **S12**. Resin-bound peptide was cleaved using **Method 7**, and the crude residue was purified by semi-preparative RP-HPLC using conditions outlined in the general method. Fractions were collected at 1 min intervals and analysed by ESI-MS and RP-HPLC. Fractions identified with the correct *m*/*z* were combined and lyophilised to afford peptide **S13** as white fluffy flakes (16.6 mg, 18.6% yield). Late-stage solution phase *C*-terminal coupling was employed to introduce the *C*-terminal aminoalcohol moiety on the peptide (16.6 mg, 0.02 mmol) dissolved in 0.5 mL DMF using **Method 8**. The crude peptide solution was purified by semi-preparative RP-HPLC in 5 × 100 μL batches using conditions outlined in the general method. Fractions were collected at 1 min intervals and analysed by ESI-MS and RP-HPLC. Fractions identified with the correct *m*/*z* were combined and lyophilised to afford trichoderin A analogue **(15)** as a white amorphous solid (3.6 mg, 3.6% overall yield based on 0.1 mmol resin loading, 98.9% purity); t_R 22.7 min; LRMS: *m*/*z* (ESI-MS) 1005.2 ($[M+H]^+$ requires 1005.4), **Figures S10-S11**. The peptide was subsequently evaluated for its *in vitro* cytotoxicity against BxPC-3 and PANC-1 human PDAC cells under both glucose-replete and glucose-deprived conditions on the same plate (**Figure S12**).

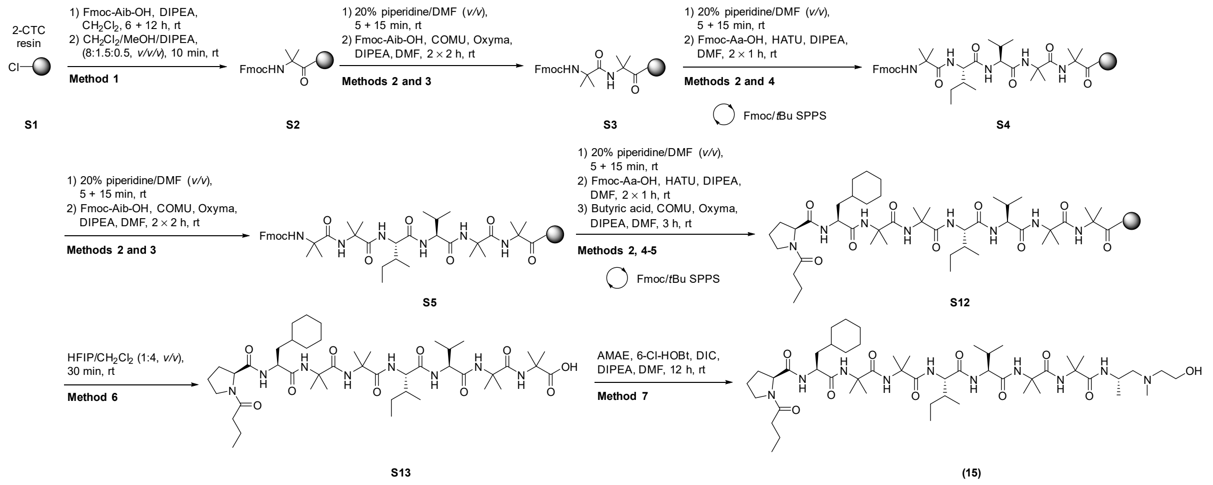

Scheme S4. Fmoc/*t*Bu SPPS of trichoderin A analogue **(15)**.

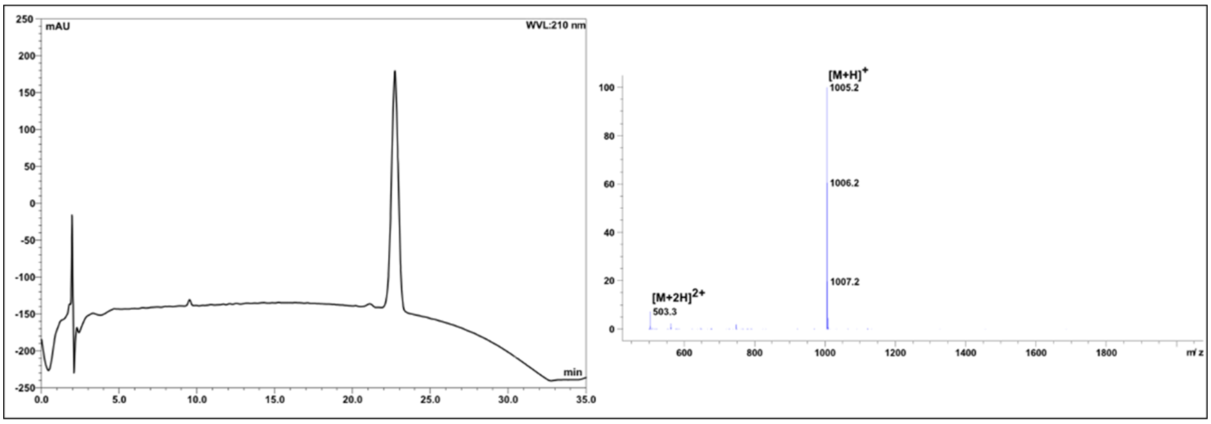

Figure S11. Analytical RP-HPLC profile of pure peptide **(15)** (*ca*. 99% as judged by peak area at 210 nm); xTerra® MS C18, 4.6 × 150 mm, 5 µm, linear gradient of 5% B to 95% B (*ca*. 3% B/min), 1 mL/min. *m/z* $[M+H]^+$ calculated: 1005.4; found: 1005.2.

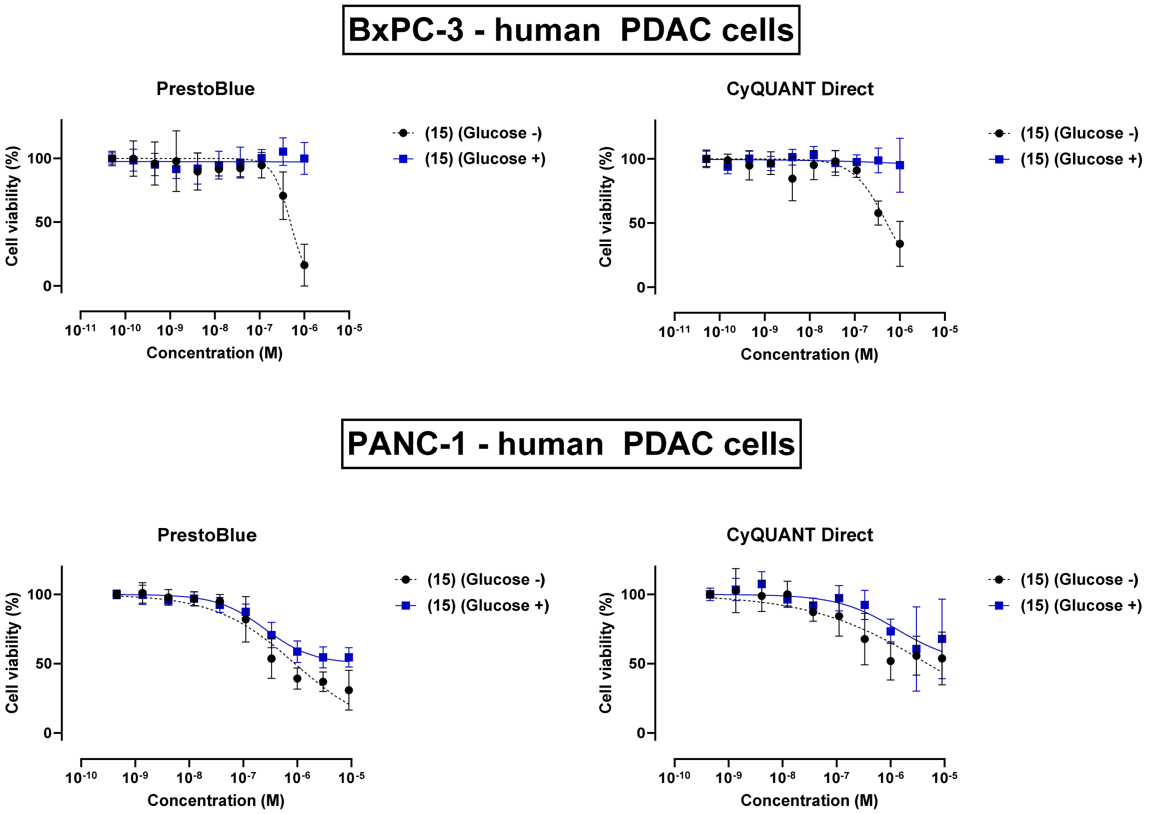

Figure S12. Comparative percent viability of BxPC-3 and PANC-1 human PDAC cells cultured under 'low glucose' (Glucose -) and 'normal glucose' (Glucose +) conditions following a 1-day exposure to trichoderin A analogue **(15)**, as judged by the PrestoBlue™ and CyQUANT™ Direct assays.

Synthesis of trichoderin A analogue (16)

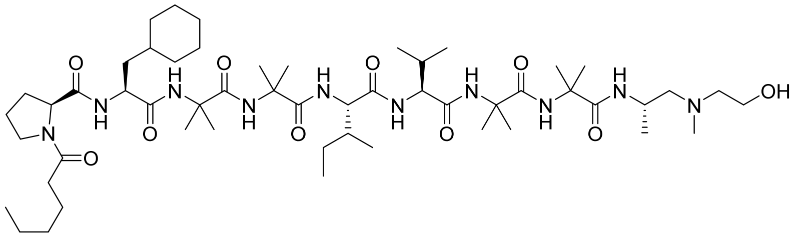

Figure S13. Chemical structure of trichoderin A analogue **(16)**.

The *C*-terminal residue Fmoc-Aib-OH was attached to 2-chlorotrityl chloride resin **S1** according to **Method 1** to form peptidyl resin **S2** (**Scheme S5**). Loading was determined to be 0.43 mmol/g by **Method 2**. The N^{α}-Fmoc-protecting group was removed using **Method 3** followed by coupling of Fmoc-Aib-OH via **Method 4** to give **S3**. The N^{α}-Fmoc-protecting group was then removed using **Method 3** followed by coupling of Fmoc-Val-OH, Fmoc-Ile-OH, and Fmoc-Aib-OH via **Method 5** to afford peptidyl resin **S4**. After removal of N^{α}-Fmoc-protecting group using **Method 3**, Fmoc-Aib-OH was coupled via **Method 4** to yield **S5**, followed by coupling of Fmoc-Cha-OH and Fmoc-Pro-OH by **Method 5**, and hexanoic acid via **Method 6** to complete the linear peptide sequence of **S14**. Resin-bound peptide was cleaved using **Method 7**, and the crude residue was purified by semi-preparative RP-HPLC using conditions outlined in the general method. Fractions were collected at 1 min intervals and analysed by ESI-MS and RP-HPLC. Fractions identified with the correct *m*/*z* were combined and lyophilised to afford peptide A15 as white fluffy flakes (13.4 mg, 14.6% yield). Late-stage solution phase *C*-terminal coupling was employed to introduce the *C*-terminal aminoalcohol moiety on the peptide (13.4 mg, 0.01 mmol) dissolved in 0.5 mL DMF using **Method 8**. The crude peptide solution was purified by semi-preparative RP-HPLC in 5 × 100 μL batches using conditions outlined in the general method. Fractions were collected at 1 min intervals and analysed by ESI-MS and RP-HPLC. Fractions identified with the correct *m*/*z* were combined and lyophilised to afford trichoderin A analogue **(16)** as a white amorphous solid (4.5 mg, 4.4% overall yield based on 0.1 mmol resin loading, 98.3% purity); t_R 24.5 min; LRMS: *m*/*z* (ESI-MS) 1033.2 ($[M+H]^+$ requires 1033.4), **Figures S13-S14**. The peptide was subsequently evaluated for its *in vitro* cytotoxicity against BxPC-3 and PANC-1 human PDAC cells under both glucose-replete and glucose-deprived conditions on the same plate (**Figure S15**).

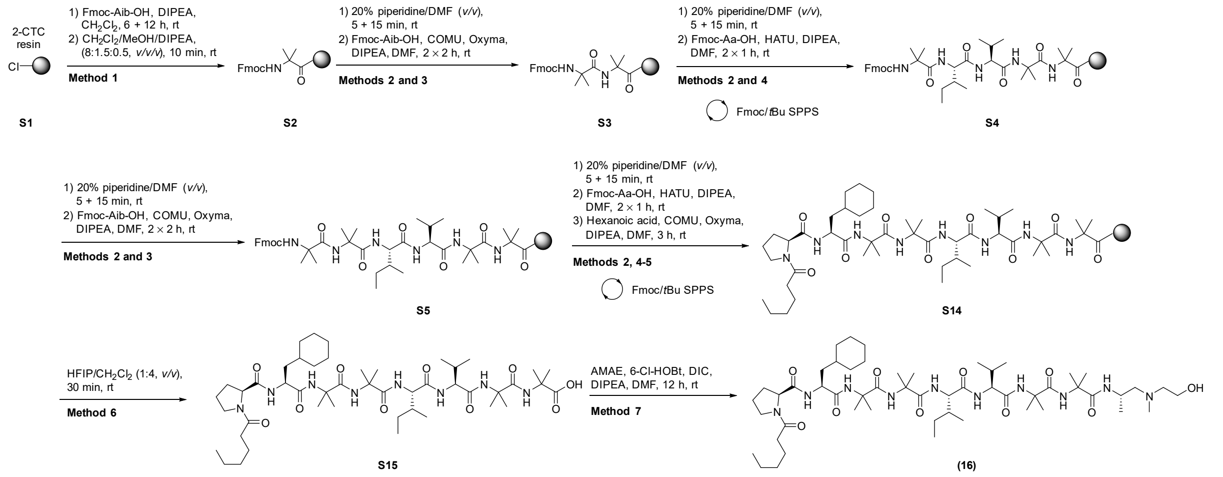

Scheme S5. Fmoc/*t*Bu SPPS of trichoderin A analogue **(16)**.

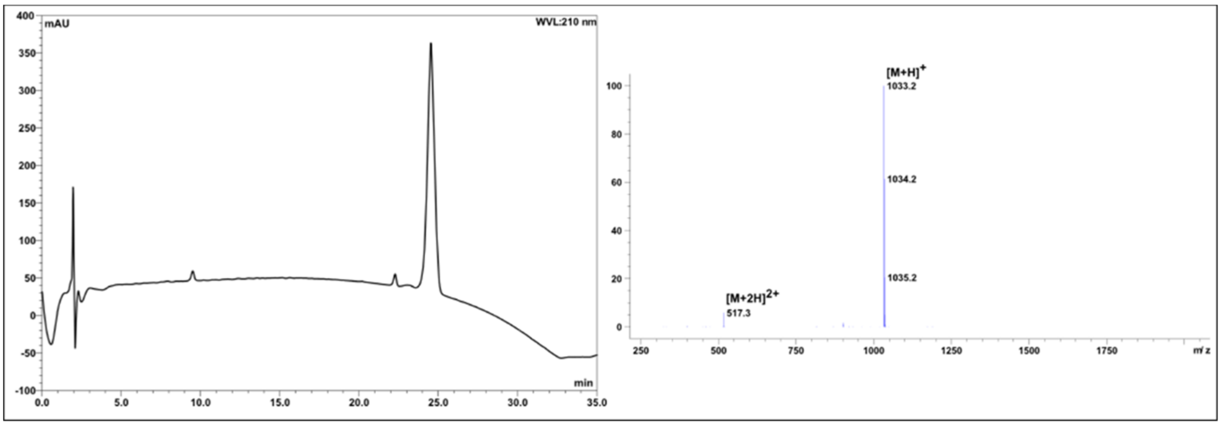

Figure S14. Analytical RP-HPLC profile of pure peptide **(16)** (*ca*. 98% as judged by peak area at 210 nm); xTerra® MS C18, 4.6 × 150 mm, 5 μm, linear gradient of 5% B to 95% B (*ca*. 3% B/min), 1 mL/min. *m*/*z* $[M+H]^+$ calculated: 1033.4; found: 1033.2.

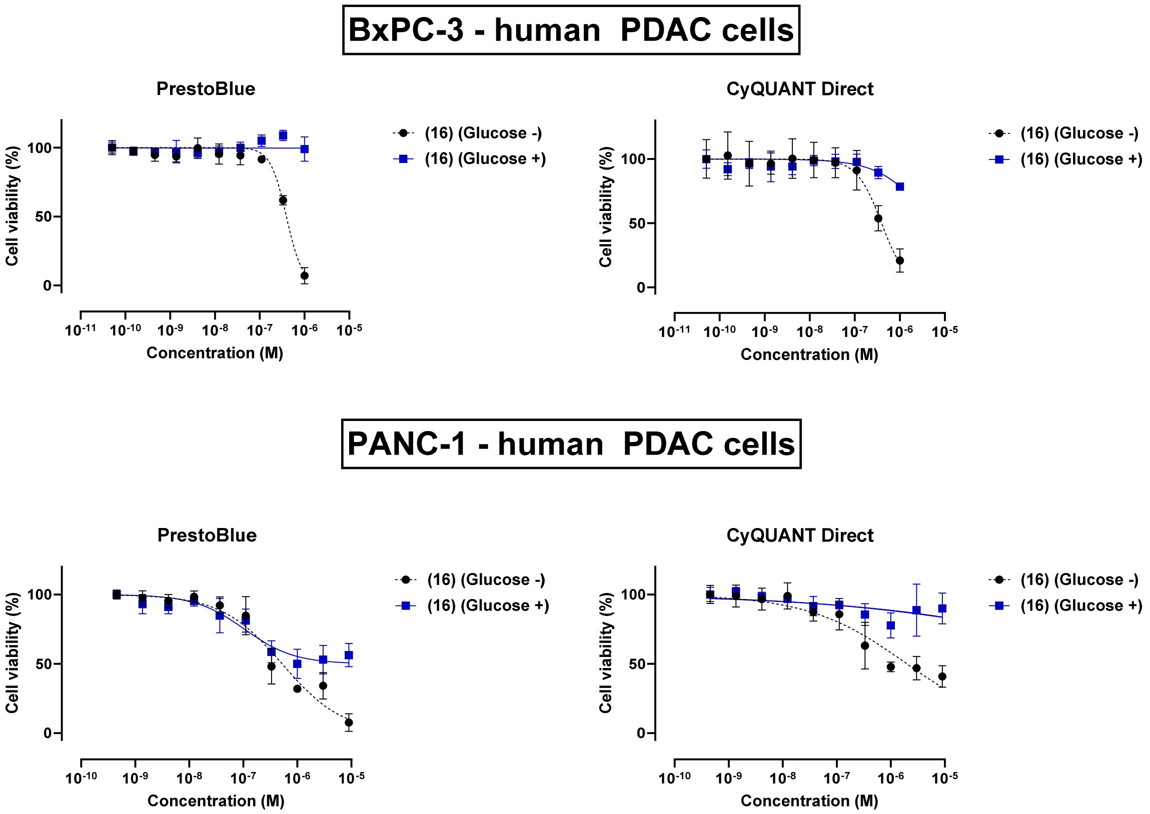

Figure S15. Comparative percent viability of BxPC-3 and PANC-1 human PDAC cells cultured under 'low glucose' (Glucose -) and 'normal glucose' (Glucose +) conditions following a 1-day exposure to trichoderin A analogue **(16)**, as judged by the PrestoBlue™ and CyQUANT™ Direct assays.

Synthesis of trichoderin A analogue (17)

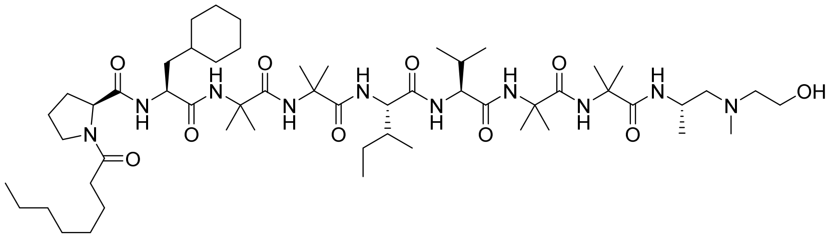

Figure S16. Chemical structure of trichoderin A analogue **(17)**.

The *C*-terminal residue Fmoc-Aib-OH was attached to 2-chlorotrityl chloride resin **S1** according to **Method 1** to form peptidyl resin **S2** (**Scheme S6**). Loading was determined to be 0.43 mmol/g by **Method 2**. The N^{α}-Fmoc-protecting group was removed using **Method 3** followed by coupling of Fmoc-Aib-OH via **Method 4** to give **S3**. The N^{α}-Fmoc-protecting group was then removed using **Method 3** followed by coupling of Fmoc-Val-OH, Fmoc-Ile-OH, and Fmoc-Aib-OH via **Method 5** to afford peptidyl resin **S4**. After removal of N^{α}-Fmoc-protecting group using **Method 3**, Fmoc-Aib-OH was coupled via **Method 4** to yield **S5**, followed by coupling of Fmoc-Cha-OH and Fmoc-Pro-OH by **Method 5**, and octanoic acid via **Method 6** to complete the linear peptide sequence of **S16**. Resin-bound peptide was cleaved using **Method 7**, and the crude residue was purified by semi-preparative RP-HPLC using conditions outlined in the general method. Fractions were collected at 1 min intervals and analysed by ESI-MS and RP-HPLC. Fractions identified with the correct *m*/*z* were combined and lyophilised to afford peptide **S17** as white fluffy flakes (15.8 mg, 16.7% yield). Late-stage solution phase *C*-terminal coupling was employed to introduce the *C*-terminal aminoalcohol moiety on the peptide (15.8 mg, 0.02 mmol) dissolved in 0.5 mL DMF using **Method 8**. The crude peptide solution was purified by semi-preparative RP-HPLC in 5 × 100 μL batches using conditions outlined in the general method. Fractions were collected at 1 min intervals and analysed by ESI-MS and RP-HPLC. Fractions identified with the correct *m*/*z* were combined and lyophilised to afford trichoderin A analogue **(17)** as a white amorphous solid (6.1 mg, 5.7% overall yield based on 0.1 mmol resin loading, >99% purity); t_R 26.2 min; LRMS: *m*/*z* (ESI-MS) 1061.3 ($[M+H]^+$ requires 1061.5), **Figures S16-S17**. The peptide was subsequently evaluated for its *in vitro* cytotoxicity against BxPC-3 and PANC-1 human PDAC cells under both glucose-replete and glucose-deprived conditions on the same plate (**Figure S18**).

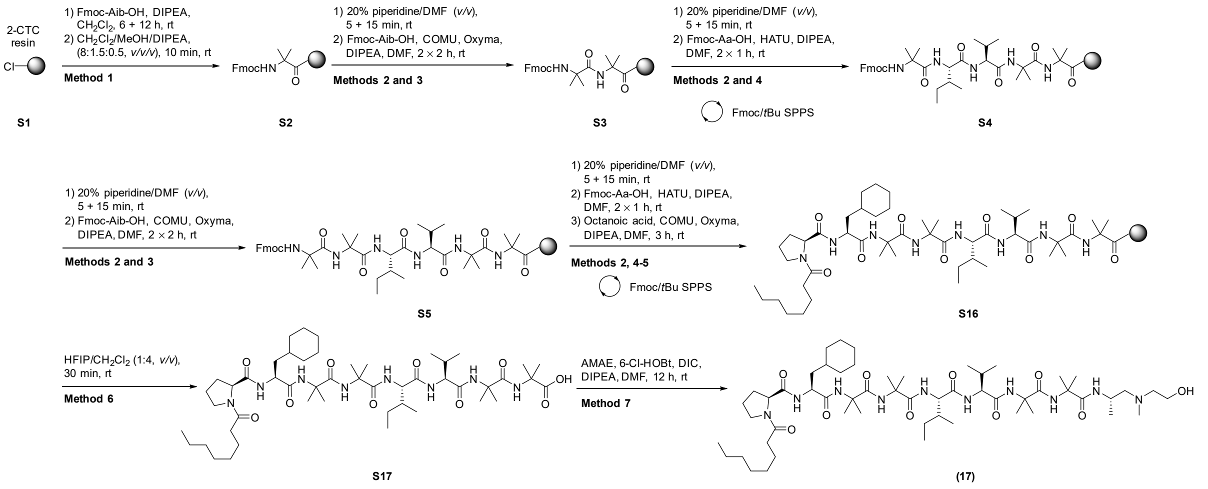

Scheme S6. Fmoc/*t*Bu SPPS of trichoderin A analogue **(17)**.

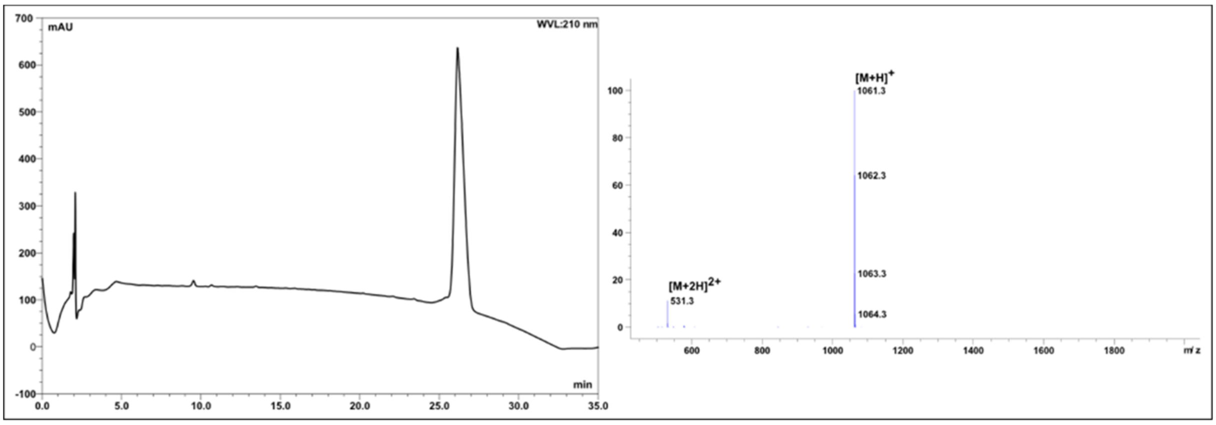

Figure S17. Analytical RP-HPLC profile of pure peptide **(17)** (*ca*. 99% as judged by peak area at 210 nm); xTerra® MS C18, 4.6 × 150 mm, 5 µm, linear gradient of 5% B to 95% B (*ca*. 3% B/min), 1 mL/min. *m*/*z* [M+H]+ calculated: 1061.5; found: 1061.3.

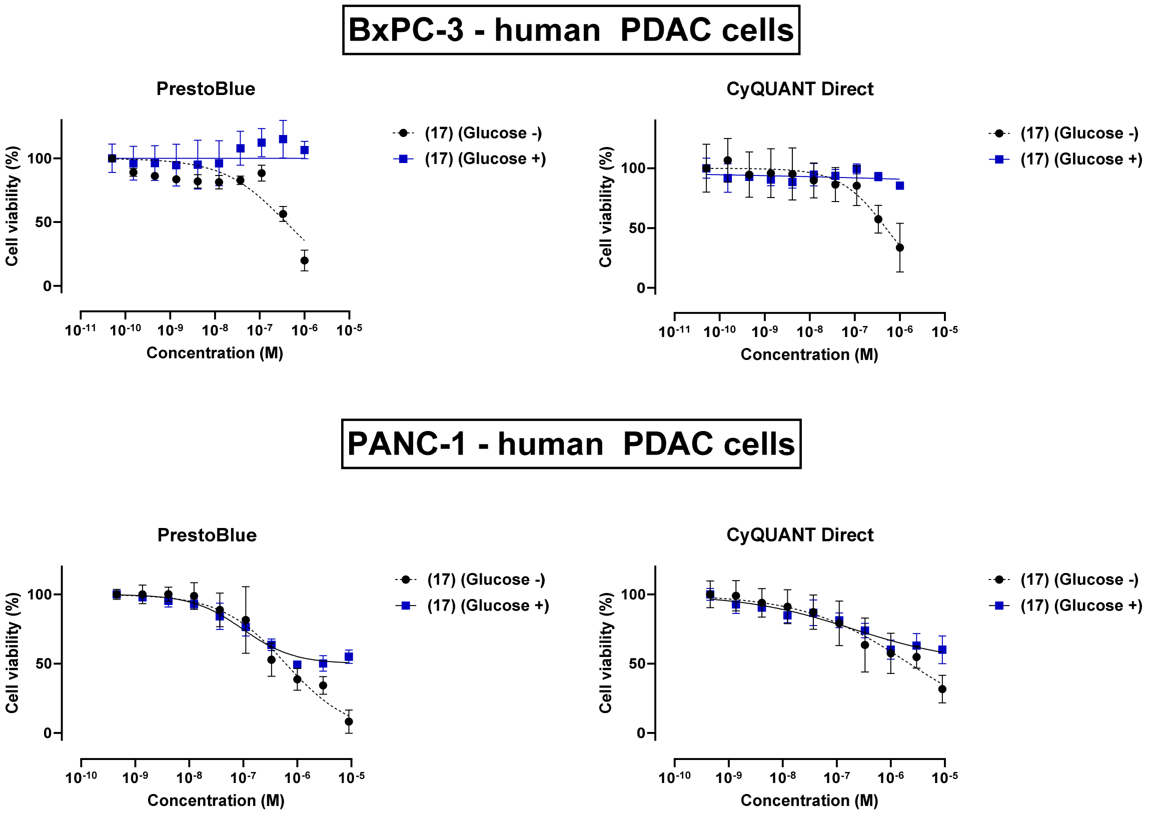

Figure S18. Comparative percent viability of BxPC-3 and PANC-1 human PDAC cells cultured under 'low glucose' (Glucose -) and 'normal glucose' (Glucose +) conditions following a 1-day exposure to trichoderin A analogue **(17)**, as judged by the PrestoBlue™ and CyQUANT™ Direct assays.

Synthesis of trichoderin A analogue (18)

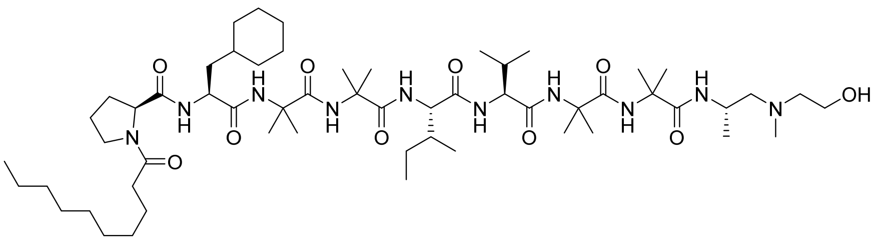

Figure S19. Chemical structure of trichoderin A analogue **(18)**.

The *C*-terminal residue Fmoc-Aib-OH was attached to 2-chlorotrityl chloride resin **S1** according to **Method 1** to form peptidyl resin **S2** (**Scheme S7**). Loading was determined to be 0.43 mmol/g by **Method 2**. The N^{α}-Fmoc-protecting group was removed using **Method 3** followed by coupling of Fmoc-Aib-OH via **Method 4** to give **S3**. The N^{α}-Fmoc-protecting group was then removed using **Method 3** followed by coupling of Fmoc-Val-OH, Fmoc-Ile-OH, and Fmoc-Aib-OH via **Method 5** to afford peptidyl resin **S4**. After removal of N^{α}-Fmoc-protecting group using **Method 3**, Fmoc-Aib-OH was coupled via **Method 4** to yield **S5**, followed by coupling of Fmoc-Cha-OH and Fmoc-Pro-OH by **Method 5**, and decanoic acid via **Method 6** to complete the linear peptide sequence of **S18**. Resin-bound peptide was cleaved using **Method 7**, and the crude residue was purified by semi-preparative RP-HPLC using conditions outlined in the general method. Fractions were collected at 1 min intervals and analysed by ESI-MS and RP-HPLC. Fractions identified with the correct *m*/*z* were combined and lyophilised to afford peptide **S19** as white fluffy flakes (25.9 mg, 26.6% yield). Late-stage solution phase *C*-terminal coupling was employed to introduce the *C*-terminal aminoalcohol moiety on the peptide (25.9 mg, 0.03 mmol) dissolved in 0.5 mL DMF using **Method 8**. The crude peptide solution was purified by semi-preparative RP-HPLC in 5 × 100 μL batches using conditions outlined in the general method. Fractions were collected at 1 min intervals and analysed by ESI-MS and RP-HPLC. Fractions identified with the correct *m*/*z* were combined and lyophilised to afford trichoderin A analogue **(18)** as a white amorphous solid (9.5 mg, 8.7% overall yield based on 0.1 mmol resin loading, 98.3% purity); t_R 28.2 min; LRMS: *m*/*z* (ESI-MS) 1089.8 ($[M+H]^+$ requires 1089.5), **Figures S19-S20**. The peptide was subsequently evaluated for its *in vitro* cytotoxicity against BxPC-3 and PANC-1 human PDAC cells under both glucose-replete and glucose-deprived conditions on the same plate (**Figure S21**).

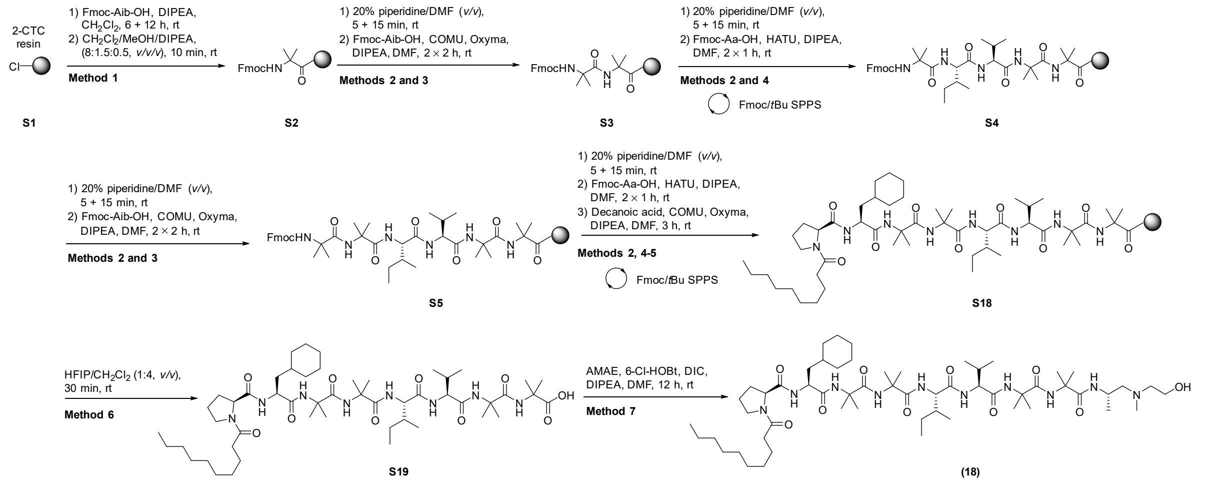

Scheme S7. Fmoc/*t*Bu SPPS of trichoderin A analogue **(18)**.

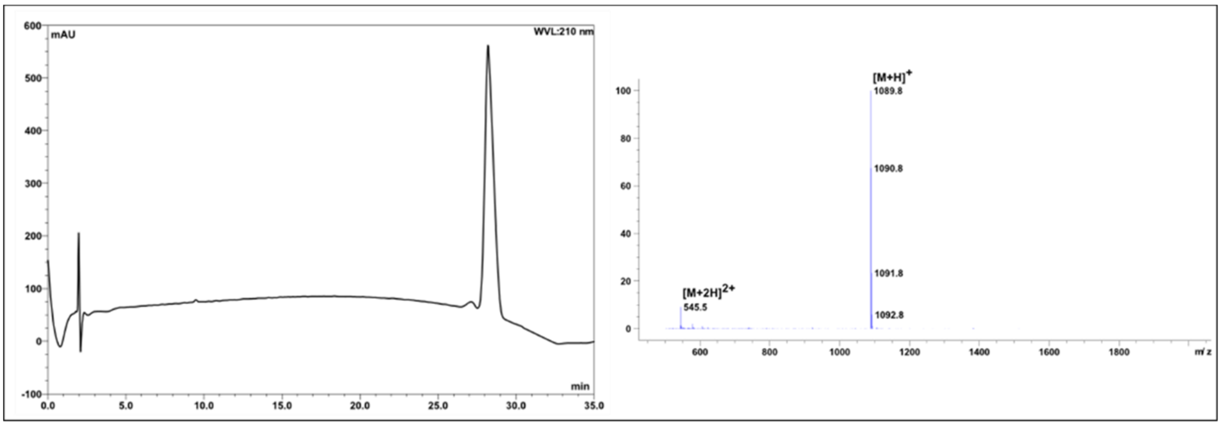

Figure S20. Analytical RP-HPLC profile of pure peptide **(18)** (*ca*. 98% as judged by peak area at 210 nm); xTerra® MS C18, 4.6 × 150 mm, 5 µm, linear gradient of 5% B to 95% B (*ca*. 3% B/min), 1 mL/min. *m*/*z* [M+H]⁺ calculated: 1089.5; found: 1089.8.

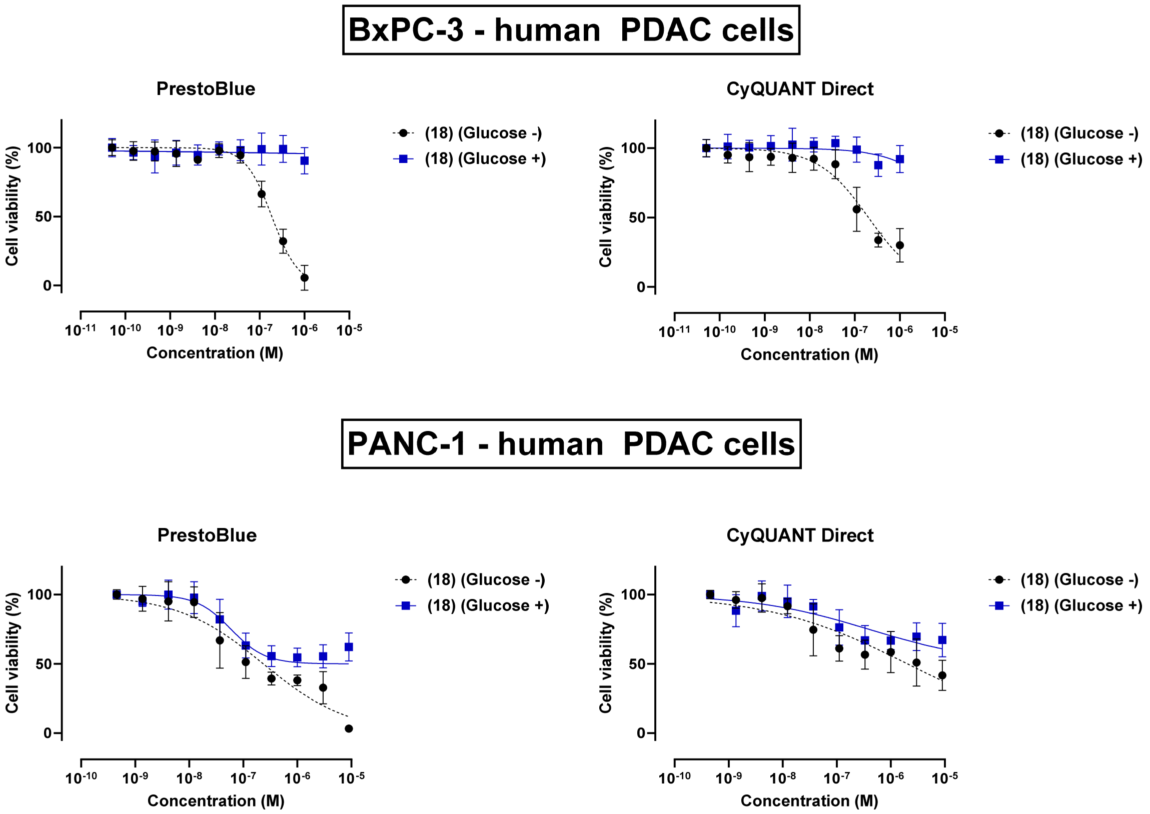

Figure S21. Comparative percent viability of BxPC-3 and PANC-1 human PDAC cells cultured under 'low glucose' (Glucose -) and 'normal glucose' (Glucose +) conditions following a 1-day exposure to trichoderin A analogue **(18)**, as judged by the PrestoBlue™ and CyQUANT™ Direct assays.

Synthesis of trichoderin A analogue (19)

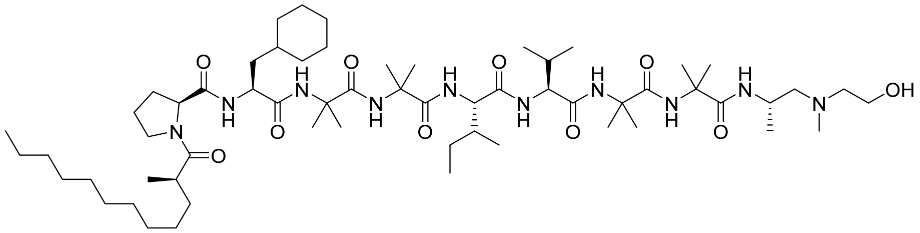

Figure S22. Chemical structure of trichoderin A analogue **(19)**.

The *C*-terminal residue Fmoc-Aib-OH was attached to 2-chlorotrityl chloride resin **S1** according to **Method 1** to form peptidyl resin **S2** (**Scheme S8**). Loading was determined to be 0.43 mmol/g by **Method 2**. The N^{α}-Fmoc-protecting group was removed using **Method 3** followed by coupling of Fmoc-Aib-OH via **Method 4** to give **S3**. The N^{α}-Fmoc-protecting group was then removed using **Method 3** followed by coupling of Fmoc-Val-OH, Fmoc-Ile-OH, and Fmoc-Aib-OH via **Method 5** to afford peptidyl resin **S4**. After removal of N^{α}-Fmoc-protecting group using **Method 3**, Fmoc-Aib-OH was coupled via **Method 4** to yield **S5**, followed by coupling of Fmoc-Cha-OH and Fmoc-Pro-OH by **Method 5**, and dodecanoic acid via **Method 6** to complete the linear peptide sequence of **S20**. Resin-bound peptide was cleaved using **Method 7**, and the crude residue was purified by semi-preparative RP-HPLC using conditions outlined in the general method. Fractions were collected at 1 min intervals and analysed by ESI-MS and RP-HPLC. Fractions identified with the correct *m*/*z* were combined and lyophilised to afford peptide **S21** as white fluffy flakes (23.2 mg, 22.5% yield). Late-stage solution phase *C*-terminal coupling was employed to introduce the *C*-terminal aminoalcohol moiety on the peptide (23.2 mg, 0.02 mmol) dissolved in 0.5 mL DMF using **Method 8**. The crude peptide solution was purified by semi-preparative RP-HPLC in 5 × 100 μL batches using outlined in the general method. Fractions were collected at 1 min intervals and analysed by ESI-MS and RP-HPLC. Fractions identified with the correct *m*/*z* were combined and lyophilised to afford trichoderin A analogue **(19)** as a white amorphous solid (12.8 mg, 11.5% overall yield based on 0.1 mmol resin loading, >99% purity); t_R 29.8 min; LRMS: *m*/*z* (ESI-MS) 1117.3 ($[M+H]^+$ requires 1117.6), **Figures S22-S23**. The peptide was subsequently evaluated for its *in vitro* cytotoxicity against BxPC-3 and PANC-1 human PDAC cells under both glucose-replete and glucose-deprived conditions on the same plate (**Figure S24**).

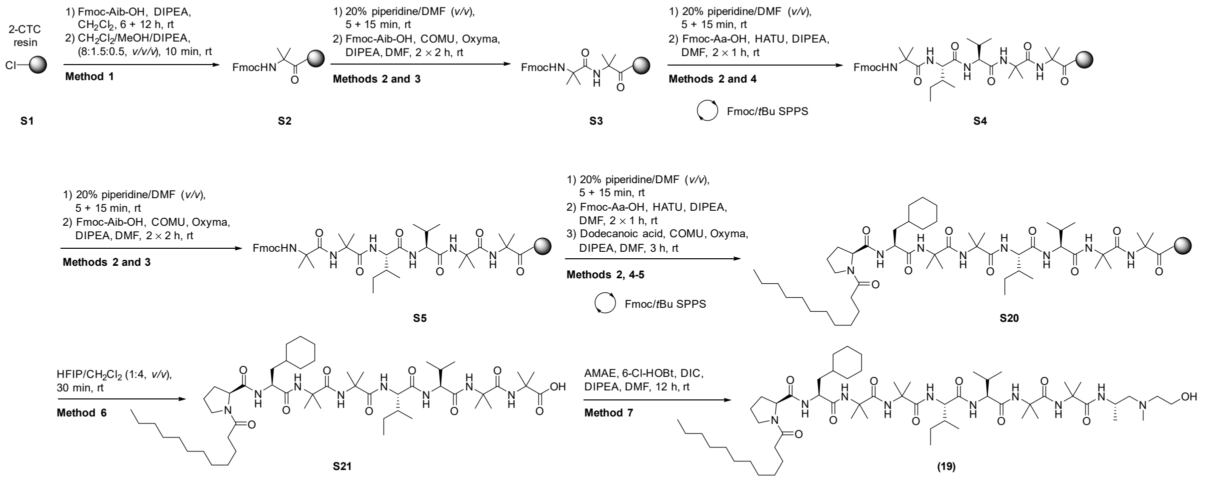

Scheme S8. Fmoc/*t*Bu SPPS of trichoderin A analogue **(19)**.

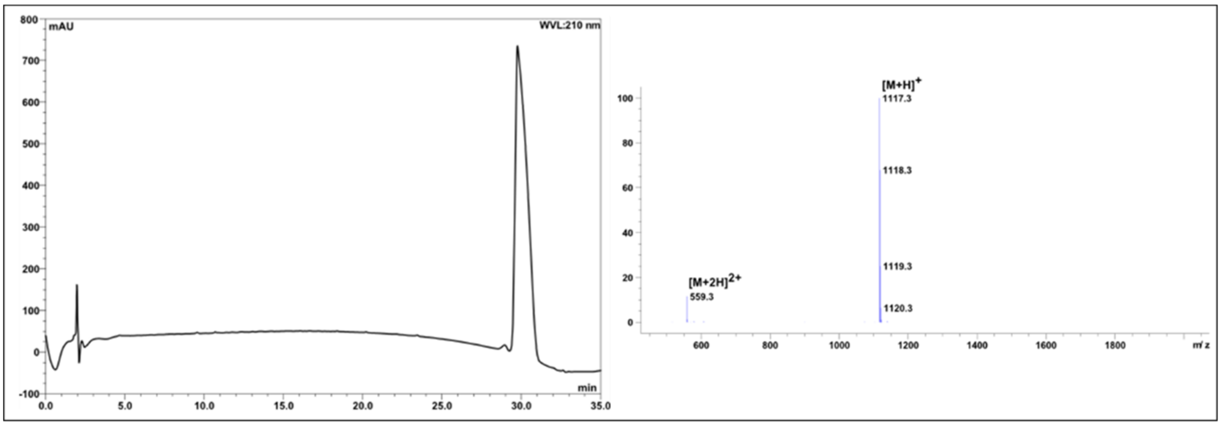

Figure S23. Analytical RP-HPLC profile of pure peptide **(19)** (*ca*. 99% as judged by peak area at 210 nm); xTerra® MS C18, 4.6 × 150 mm, 5 µm, linear gradient of 5% B to 95% B (*ca*. 3% B/min), 1 mL/min. *m*/*z* $[M+H]^+$ calculated: 1117.6; found: 1117.3.

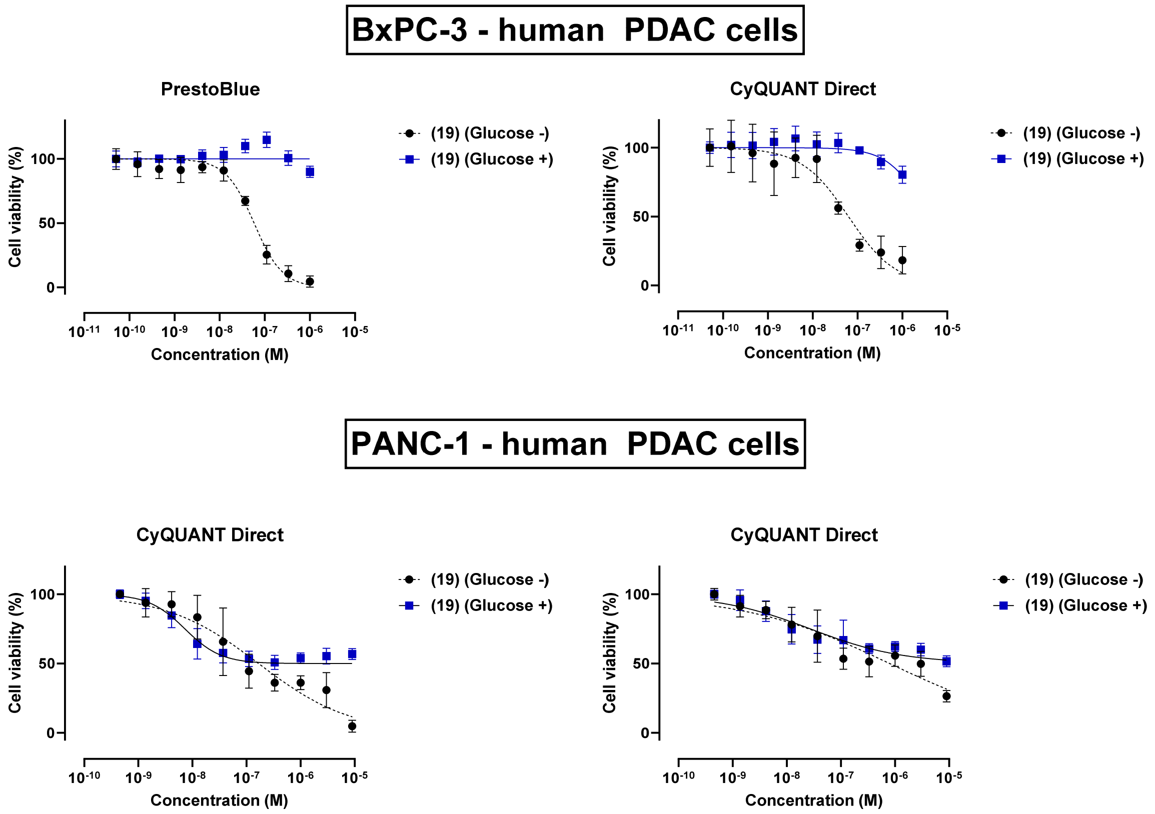

Figure S24. Comparative percent viability of BxPC-3 and PANC-1 human PDAC cells cultured under 'low glucose' (Glucose -) and 'normal glucose' (Glucose +) conditions following a 1-day exposure to trichoderin A analogue **(19)**, as judged by the PrestoBlue™ and CyQUANT™ Direct assays.

References

(1) Kavianinia, I.; Kunalingam, L.; Harris, P. W. R.; Cook, G. M.; Brimble, M. A. Total Synthesis and Stereochemical Revision of the Anti-Tuberculosis Peptaibol Trichoderin A. *Org. Lett.* **2016**, *18* (15), 3878–3881. https://doi.org/10.1021/acs.orglett.6b01886.
